# Prediction of remission and low disease activity in disease-modifying anti-rheumatic drug-refractory patients with rheumatoid arthritis treated with golimumab

**DOI:** 10.1093/rheumatology/kew179

**Published:** 2016-04-25

**Authors:** Nathan Vastesaeger, Abraham Garcia Kutzbach, Howard Amital, Karel Pavelka, María Alicia Lazaro, Robert J. Moots, Jürgen Wollenhaupt, Cristiano A. F. Zerbini, Ingrid Louw, Bernard Combe, Andre Beaulieu, Hendrik Schulze-Koops, Bhaskar Dasgupta, Bo Fu, Susan Huyck, Haoling H. Weng, Marinella Govoni, Patrick Durez

**Affiliations:** ^1^Department of Medical Affairs, MSD Danmark ApS, Ballerup, Denmark; ^2^Department of Rheumatology, AGAR Francisco Marroquin University, Guatemala City, Guatemala; ^3^Department of Internal Medicine B and Research Center for Autoimmune Diseases, Sheba Medical Center, Tel-Hashomer, Israel; ^4^Institute of Rheumatology and Clinic of Rheumatology, Charles University, Prague, Czech Republic; ^5^Instituto de Asistencia Reumatologica Integral, Buenos Aires, Argentina; ^6^Department of Rheumatology, Clinical Sciences Centre, University Hospital Aintree, Liverpool, UK; ^7^Department of Rheumatology, Klinik für Rheumatologie, Schön Klinik Hamburg-Eilbek, Hamburg, Germany; ^8^Department of Rheumatology, Centro Paulista de Investigação Clinica, São Paulo, Brazil; ^9^Panorama Medical Centre, Cape Town, South Africa; ^10^Departement de Rhumatologie, Hôpital Lapeyronie, Montpellier University Hospital, Montpellier, France; ^11^Centre de Rhumatologie St-Louis, Québec, Canada; ^12^Division of Rheumatology and Clinical Immunology, Department of Medicine IV, University of Munich, Munich, Germany; ^13^Department of Rheumatology, Southend University Hospital, Westcliff-on-Sea, Essex, UK; ^14^Department of Biostatistics; ^15^Clinical Development, Merck & Co, Inc., Kenilworth, NJ, USA; ^16^Department of Immunology, MSD Italy, Global Medical Affairs, Rome, Italy; ^17^Department of Rheumatology, Université Catholique de Louvain, Brussels, Belgium

**Keywords:** rheumatoid arthritis, predictors of response, remission, tumour necrosis factor, biologic

## Abstract

**Objective.** To create a tool to predict probability of remission and low disease activity (LDA) in patients with RA being considered for anti-TNF treatment in clinical practice.

**Methods.** We analysed data from GO-MORE, an open-label, multinational, prospective study in biologic-naïve patients with active RA (DAS28-ESR ⩾3.2) despite DMARD therapy. Patients received 50 mg s.c. golimumab (GLM) once monthly for 6 months. In secondary analyses, regression models were used to determine the best set of baseline factors to predict remission (DAS28-ESR <2.6) at month 6 and LDA (DAS28-ESR ⩽3.2) at month 1.

**Results.** In 3280 efficacy-evaluable patients, of 12 factors included in initial regression models predicting remission or LDA, six were retained in final multivariable models. Greater likelihood of LDA and remission was associated with being male; younger age; lower HAQ, ESR (or CRP) and tender joint count (or swollen joint count) scores; and absence of comorbidities. In models predicting 1-, 3- and 6-month LDA or remission, area under the receiver operating curve was 0.648–0.809 (R^2 ^= 0.0397–0.1078). The models also predicted 6-month HAQ and EuroQoL-5-dimension scores. A series of matrices were developed to easily show predicted rates of remission and LDA.

**Conclusion.** A matrix tool was developed to show predicted GLM treatment outcomes in patients with RA, based on a combination of six baseline characteristics. The tool could help provide practical guidance in selection of candidates for anti-TNF therapy.

Rheumatology key messages

- A regression model incorporating baseline characteristics predicted golimumab treatment outcomes in patients with RA.
- A practical tool was developed to help select RA patients for anti-tumour necrosis factor therapy.

## Introduction

To make best use of resources for biologic treatment in patients with RA, it would be useful to identify a set of predictors that enable selection of patients who will benefit most from such treatment and avoid treatment of patients who are unlikely to respond. Several studies have evaluated predictors of outcomes during anti-TNF treatment (for a review, see [1, 2]). One limitation of some of these studies [3] is that the predictive capacity of single predictors is low (*vs* combinations of predictors), and they are less useful when making practice decisions for individual patients.

Although there is variability in which factors have predictive ability, some of the baseline characteristics that have been found to predict anti-TNF outcomes include baseline age (e.g. [4, 5]), smoking (e.g. [6–8]), gender (e.g. [6, 9]), disease activity (e.g. [10, 11]) and functional ability (e.g. [4]). Predictors that are significant across studies may depend on factors such as the patient population, type of treatment, the outcome being evaluated and whether the outcome is a state measure or an improvement measure.

Current EULAR recommendations emphasize low disease activity (LDA) or remission as the treatment goal in RA and advocate the use of poor prognostic factors to guide treatment decisions [12]. If a patient does not attain remission or LDA with DMARDs and if poor prognostic factors are present (e.g. high disease activity, RF positivity and CCP antibodies, erosive disease), EULAR recommendations suggest the addition of a biologic treatment. However, poor prognostic factors such as high baseline disease activity have also been shown to be associated with poorer anti-TNF treatment outcomes; that is, patients who begin anti-TNF treatment with high disease activity have been reported to be less likely to achieve remission or LDA than patients who begin treatment with more moderate disease activity (e.g. [11, 13]). This adds complexity to clinical decisions balancing risks and benefits to determine which patients will benefit most from anti-TNF treatment.

The goal of these analyses was to develop a tool that can be used to assist in decision making to optimize treatment goal attainment in patients with RA who have failed DMARD treatment. The tool identifies groups of patients who would most likely benefit from golimumab (GLM) therapy and presents findings in a form that is simple and can be used in daily clinical practice.

## Methods

### Design and patients

Analysis of associations between baseline characteristics and outcomes of treatment was a key secondary objective of the GO-MORE trial. GO-MORE was an open-label, prospective study of add-on treatment with GLM in patients with active RA despite DMARD treatment in 40 countries (protocol P06129; NCT00975130). Details of the study procedures have been previously reported [13] and are only briefly described here. The GO-MORE study received approval from appropriate research ethics committees in each country and was conducted in accordance with the Declaration of Helsinki and standards of good clinical research practice. All patients consented to participate in the GO-MORE study. The analysis for this study did not require a separate approval. Data were collected from 29 October 2009 to 21 July 2011. Patients in GO-MORE were biologic-naïve with active RA (DAS28-ESR ⩾3.2) despite DMARD therapy and had no contraindications for TNF inhibitor treatment.

### Study procedures

In the first 6 months of GO-MORE, all patients received monthly s.c. GLM 50 mg administered by autoinjector and had efficacy and safety assessments at months 1, 3 and 6. At month 6, patients who had good or moderate EULAR response but were not in remission were able to continue to part 2 of the study, an extension phase that is described in length elsewhere [13].

### Statistical analyses

A sequence of steps was used to develop an optimal model to predict remission. The main outcomes to be predicted were DAS28-ESR LDA at the end of month 1 (after one injection) and remission at the end of month 6. The model’s ability to predict DAS28-ESR remission, DAS28-ESR LDA, Simple Disease Activity Index (SDAI) remission (SDAI ⩽3.3) and LDA (SDAI ⩽11 = LDA), and DAS28 based on CRP (cut-off criteria: <2.6 and ⩽3.2) at different time points during treatment was also explored.

Baseline predictors included in the initial univariate analyses were gender, age, disease duration, smoking status, comorbidities, number of previously failed DMARDs, 28-joint tender joint count (TJC28), 28-joint swollen joint count (SJC28), patient global assessment of disease activity (measured on a 100-mm visual analogue scale), HAQ score, MTX dose and log ESR. Characteristics that predicted DAS28-ESR remission at month 6 and LDA at month 1 at the P < 0.10 level were retained and used in multivariable models. Stepwise selection was used as a sensitivity analysis to confirm the factors selected for the model.

To determine whether TJC and SJC or ESR and CRP could be used interchangeably with no loss in predictive power, multivariable models switching out these components were compared. Area under the receiver operating characteristic curve (AUC-ROC) analysis and R^2^ were used to evaluate the models.

To create the matrix tool, continuous variables were transformed to categorical variables, using tertiles or quartiles. Models with three- and four-level categories were evaluated using AUC-ROC analysis. The predicted LDA/remission rates were displayed in an easily readable colour-coded matrix.

Additional analyses were performed to explore the model’s usefulness and limitations. For these additional analyses, patients were divided into subgroups of DAS28 predicted remission at 6 months (patients predicted to have <10% chance of DAS28 remission, 10 to <20%, 20 to < 30%, 30 to <40%, 40 to < 50% and ⩾50% chance of DAS28 remission). First, the associations between the predictors and outcomes of physical function (HAQ) and quality of life [EuroQol-5-dimension (EQ-5)] were explored. For patients in each prediction subgroup, median and interquartile ranges for DAS28, HAQ and EQ-5D at baseline and 6 months and the change in value from baseline to 6 months were calculated. Next, the association between the predictors and DAS28 improvement (as opposed to DAS28 disease state) over 6 months was investigated. Finally, the associations between the predictors and EULAR response and the associations between the predictors and ACR criteria for response were evaluated.

## Results

### Disposition and baseline characteristics

Of 3366 patients enrolled in GO-MORE, 3280 were included in the efficacy evaluable population. The patient disposition and baseline characteristics are fully reported in [13]. A summary of baseline characteristics is shown in Table 1. A majority of patients were female (82.8%, 2716/3280) and the mean (s.d.) age was 52.3 (12.8) years. A majority of patients 78.7% (2572/3280) had high disease activity (DAS28-ESR >5.1) at baseline; the remaining patients had moderate disease activity (DAS28-ESR of 3.2–5.1) at baseline. At least one comorbidity was reported by 76.2% of patients (2499/3280). See supplementary Table S1, available at *Rheumatology* Online, for a list of the comorbidities that were reported in ⩾2% of patients.

**Table 1:** Demographics and baseline characteristics of patients in the efficacy population of GO-MORE

Patient characteristics	n = 3280
Demographic characteristics	
Female, n (%)	2716 (82.8)
Age, median (min, max), years	53.0 (18, 88)
Disease characteristics	
Disease duration, years	n = 3279
Median (min, max)	4.9 (0.01, 56.6)
TJC28, mean (s.d.)	13.0 (6.81)
SJC28, mean (s.d.)	9.6 (5.56)
DAS28-ESR	n = 3270
Moderate disease activity (3.2–5.1), n (%)	698 (21.3)
High disease activity (>5.1), n (%)	2572 (78.7)
Mean (s.d.)	5.97 (1.095)
DAS28-CRP	n = 3236
Mean (sd)	5.41 (0.998)
CRP, mg/l	n = 3236
Mean (s.d.)	14.48 (20.376)
ESR, mm/h	n = 3280
Mean (s.d.)	34.9 (24.64)
Anti-CCP	n = 3225
Positive (≥20 U/ml), n (%)	2318 (71.9)
RF	n = 3234
Positive (≥15 IU/ml), n (%)	2344 (72.5)
HAQ-DI, mean (s.d.)	1.44 (0.67)

Table adapted from Combe B, Dasgupta B, Louw I, *et al.* Efficacy and safety of golimumab as add-on therapy to disease-modifying antirheumatic drugs: results of the GO-MORE study. Ann Rheum Dis 2014;73:1477–86 [13]. with permission © (2004) BMJ publishing group. SJC28: joint swollen joint count 28; TJC28, joint tender joint count 28; n: number.

### Regression model for prediction of remission and LDA

At the end of month 6, 23.9% of patients had achieved DAS28-ESR remission and 37.4% achieved LDA; at the end of month 1, 16.6% of patients had achieved LDA [13]. Initial univariate analyses narrowed the set of factors that were candidates for the multivariable models predicting remission and LDA. Factors retained [those that had significant relationships (P < 0.10) with DAS28 remission at month 6 and LDA at month 1] were analysed in a multivariable model predicting DAS28-ESR remission at month 6 (supplementary Table S2, available at *Rheumatology* Online). Factors retained after this step were gender, HAQ, presence of comorbidities, age, TJC and ESR. Smoking was associated with remission at 6 months but not with LDA at 1 month, and therefore was not retained.

Overall, the predictive value of the model (Table 2) was slightly weakened by replacing TJC with SJC or by replacing CRP with ESR in the models predicting DAS28. The pattern of results was similar for models predicting SDAI and DAS28-CRP outcomes at months 1, 3 and 6. Prediction of outcomes at month 1 was slightly better than for outcomes at month 6.

**Table 2:** Prediction of multiple disease activity outcomes with three different sets of baseline factors

Outcome predicted	Baseline predictor set^a^	Month 1		Month 3		Month 6	
		AUC	R^2^	AUC	R^2^	AUC	R^2^
DAS28-ESR remission	With TJC, ESR	0.809	0.0954	0.738	0.1002	0.717	0.1078
	With SJC, CRP	0.729	0.0521	0.694	0.0696	0.687	0.0815
	With TJC, CRP	0.758	0.0660	0.707	0.0788	0.702	0.0949
DAS28-ESR LDA	With TJC, ESR	0.795	0.1565	0.734	0.1372	0.710	0.1261
	With SJC, CRP	0.724	0.0937	0.682	0.0874	0.665	0.0807
	With TJC, CRP	0.757	0.1202	0.702	0.1057	0.690	0.1052
SDAI remission	With TJC, ESR	0.708	0.0168	0.664	0.0282	0.655	0.0394
	With SJC, CRP	0.703	0.0153	0.648	0.0226	0.648	0.0353
	With TJC, CRP	0.706	0.0158	0.663	0.0288	0.658	0.0409
SDAI LDA	With TJC, ESR	0.707	0.0145	0.661	0.0266	0.660	0.0397
	With SJC, CRP	0.705	0.0144	0.649	0.0224	0.651	0.0352
	With TJC, CRP	0.701	0.0142	0.662	0.0281	0.664	0.0418
DAS28-CRP <2.6	With TJC, ESR	0.738	0.0777	0.687	0.0785	0.674	0.0820
	With SJC, CRP	0.698	0.0536	0.658	0.0570	0.661	0.0711
	With TJC, CRP	0.737	0.0780	0.687	0.0795	0.683	0.0924
DAS28-CRP ≤3.2	With TJC, ESR	0.751	0.1448	0.700	0.1152	0.683	0.0995
	With SJC, CRP	0.712	0.1063	0.681	0.0954	0.661	0.0791
	With TJC, CRP	0.753	0.1473	0.705	0.1209	0.689	0.1071

^a^All factor sets include continuous HAQ and categorical gender and comorbidity. Inclusion of ESR, CRP, TJC and SJC varied as indicated. TJC, SJC, ESR and CRP were all continuous variables. CRP was used in logarithm scale. AUC: area under the curve; LDA: low disease activity; SDAI: Simple Disease Activity Index; SJC: swollen joint count; TJC: tender joint count.

To translate the data into the prediction matrix tool, continuous predictor variables (age, ESR and TJC) had to be transformed to categorical variables with either three or four levels. AUC and R^2^ values for models with the continuous *vs* categorical variables indicated that little predictive power was lost moving from continuous to categorical variables (data not shown). Categorical variables with three levels were selected for all but age because this resulted in a simpler matrix tool.

### Prediction matrix tool

**Fig. 1:**
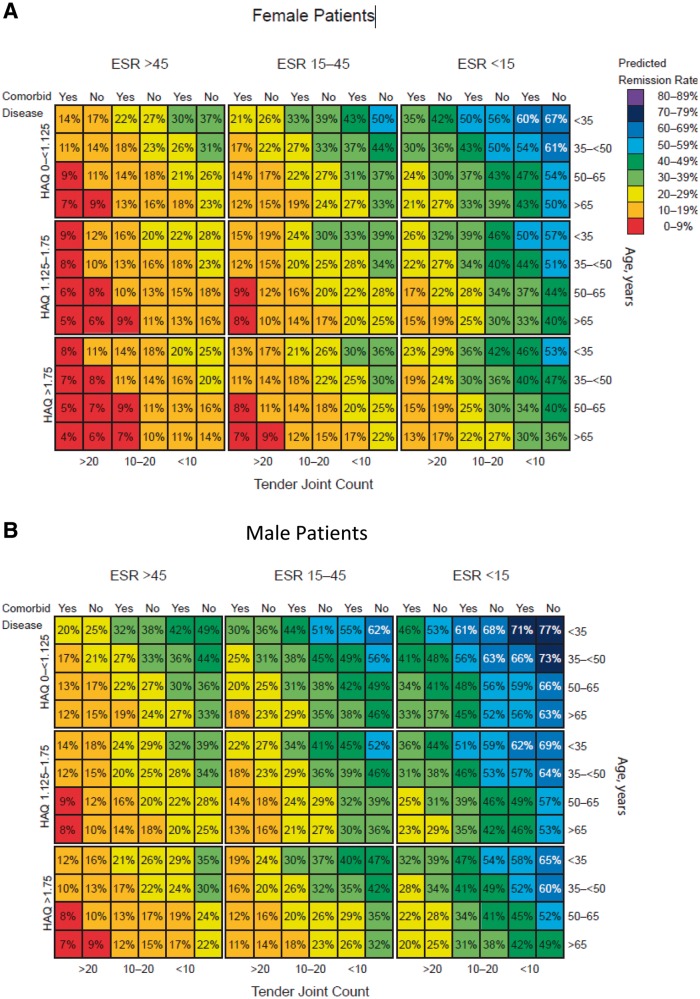

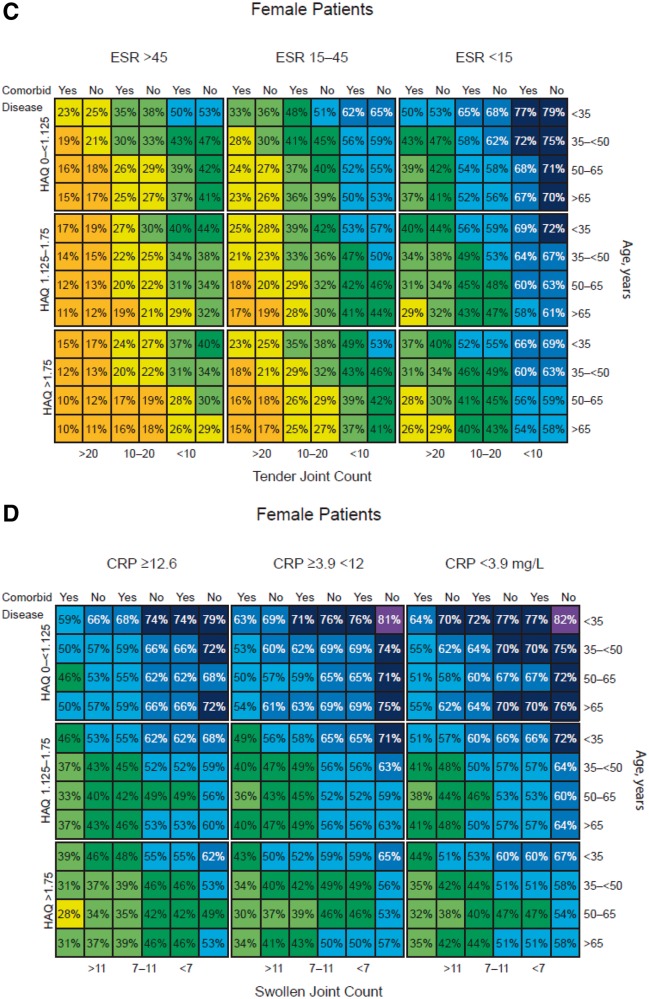
Matrix tool predicting outcomes of golimumab treatment at month 6 The model estimates outcomes at month 6 for each combination of predictor variables. Predicted rates shown for DAS28-ESR remission in female (**A**) and male (**B**) patients at 6 months, DAS28-ESR LDA in female patients at 6 months (**C**), and SDAI low disease activity in female patients using CRP instead of ESR as a predictor at 6 months (**D**). LDA: low disease activity; SDAI: Simple Disease Activity Index.

Predicted remission and LDA rates from the final multivariable models were used to create a series of matrix tools, as shown in Fig. 1 and supplementary Fig. S1, available at *Rheumatology* Online. Figures for males and females were generated separately (the impact of each predictor was similar in each gender group, but males had better outcomes overall). Separate models were generated to predict DAS28 remission and LDA, and for SDAI remission and LDA. In addition, separate models were created for use with ESR *vs* CRP as the inflammatory marker.

Each matrix shows the predicted remission or LDA rate for every combination of the six baseline factors, representing a total of 432 different RA subpopulations. Across all models, the highest remission rates are in the cells at the upper right and the lowest rates in the cells at the lower left. The colour codes have been added for easier visual perception of the matrix, with each colour representing an estimated range of 10% likelihood of remission or LDA; warmer colours (e.g. red, yellow) indicate worse predicted outcomes and cooler colours (e.g. blue, purple) indicate better predicted outcomes. Odds ratios (ORs) associated with each factor are shown in Table 3. Low baseline TJC and ESR were the strongest predictors in the model. In addition, higher remission rates are predicted for patients who are male, have no comorbidities, are younger in age and have lower baseline HAQ.

**Table 3:** Odds ratios for associations of individual predictor variables with DAS28-ESR remission and LDA at months 1, 3 and 6

	OR (95% CI)		
Variable	Month 1	Month 3	Month 6
	DAS28-ESR remission		
ESR			
<15 *vs* ≥45	9.18 (5.62, 15.01)	4.71 (3.45, 6.42)	3.43 (2.66, 4.41)
≥15 to < 45 *vs* ≥45	2.62 (1.60, 4.29)	2.20 (1.64, 2.94)	1.68 (1.33, 2.11)
Gender			
Male *vs* female	1.58 (1.16, 2.15)	1.73 (1.37, 2.19)	1.64 (1.33, 2.02)
HAQ			
0 to 1.125 *vs* ≥1.75	1.70 (1.18, 2.45)	1.40 (1.09, 1.80)	1.79 (1.44, 2.22)
≥1.125 to < 1.75 *vs* ≥1.75	1.48 (0.99, 2.19)	1.19 (0.91, 1.56)	1.20 (0.95, 1.51)
Age, years			
<35 *vs* ≥65	1.71 (1.02, 2.87)	2.00 (1.33, 2.99)	1.92 (1.35, 2.73)
≥35 to < 50 *vs* ≥65	1.15 (0.74, 1.78)	1.59 (1.14, 2.21)	1.54 (1.16, 2.04)
≥50 to < 65 *vs* ≥65	0.88 (0.57, 1.34)	1.23 (0.90, 1.70)	1.14 (0.88, 1.49)
Comorbidities			
No *vs* yes	1.51 (1.11, 2.04)	1.44 (1.15, 1.81)	1.34 (1.10, 1.65)
TJC			
<10 *vs* ≥20	5.12 (2.92, 9.00)	2.81 (2.02, 3.92)	2.87 (2.14, 3.84)
≥10 to < 20 *vs* ≥20	2.09 (1.18, 3.70)	1.43 (1.03, 1.99)	1.85 (1.39, 2.45)
	DAS28-ESR low disease activity		
ESR			
<15 *vs* ≥45	5.92 (4.32, 8.11)	4.35 (3.40, 5.55)	3.34 (2.67, 4.18)
≥15 to < 45 *vs* ≥45	2.13 (1.58, 2.87)	1.78 (1.44, 2.21)	1.67 (1.38, 2.02)
Gender			
Male *vs* female	1.65 (1.30, 2.10)	1.46 (1.18, 1.80)	1.44 (1.18, 1.76)
HAQB			
0 to 1.125 *vs* ≥1.75	1.79 (1.38, 2.32)	1.59 (1.30, 1.96)	1.68 (1.39, 2.03)
≥1.125 to < 1.75 *vs* ≥1.75	1.34 (1.01, 1.79)	1.25 (1.00, 1.56)	1.16 (0.95, 1.41)
Age, years			
<35 *vs* ≥65	1.58 (1.07, 2.33)	1.47 (1.05, 2.06)	1.65 (1.20, 2.26)
≥35 to < 50 *vs* ≥65	1.07 (0.78, 1.46)	1.24 (0.96, 1.61)	1.27 (1.00, 1.62)
≥50 to < 65 *vs* ≥65	0.82 (0.61, 1.11)	1.02 (0.80, 1.30)	1.07 (0.86, 1.34)
Comorbidities			
No *vs* yes	1.49 (1.18, 1.89)	1.36 (1.11, 1.65)	1.15 (0.95, 1.39)
TJC			
<10 *vs* ≥20	6.13 (4.13, 9.08)	3.65 (2.77, 4.81)	3.35 (2.62, 4.28)
≥10 to < 20 *vs* ≥20	2.04 (1.37, 3.03)	1.85 (1.42, 2.42)	1.85 (1.46, 2.33)

EQ-5D: EuroQol 5-dimension; HAQB: health assessment questionnaire at baseline; LDA: low disease activity; OR: odds ratio; TJC: tender joint count.

### Additional analyses to explore the matrix model’s usefulness and limitations

The factors in the model built to predict DAS28 remission or LDA also were associated with attainment of meaningful EQ-5D and HAQ cut-offs (supplementary Table S3, available at *Rheumatology* Online). Of all the factors included in the model, baseline HAQ was most strongly related to attainment of cut-off levels for EQ-5D (⩾0.7 and ⩾0.8) and HAQ (<0.5) at month 6 (ORs from 2.38 to 6.55 for the lowest *vs* highest HAQ score categories).

**Fig. 2:**
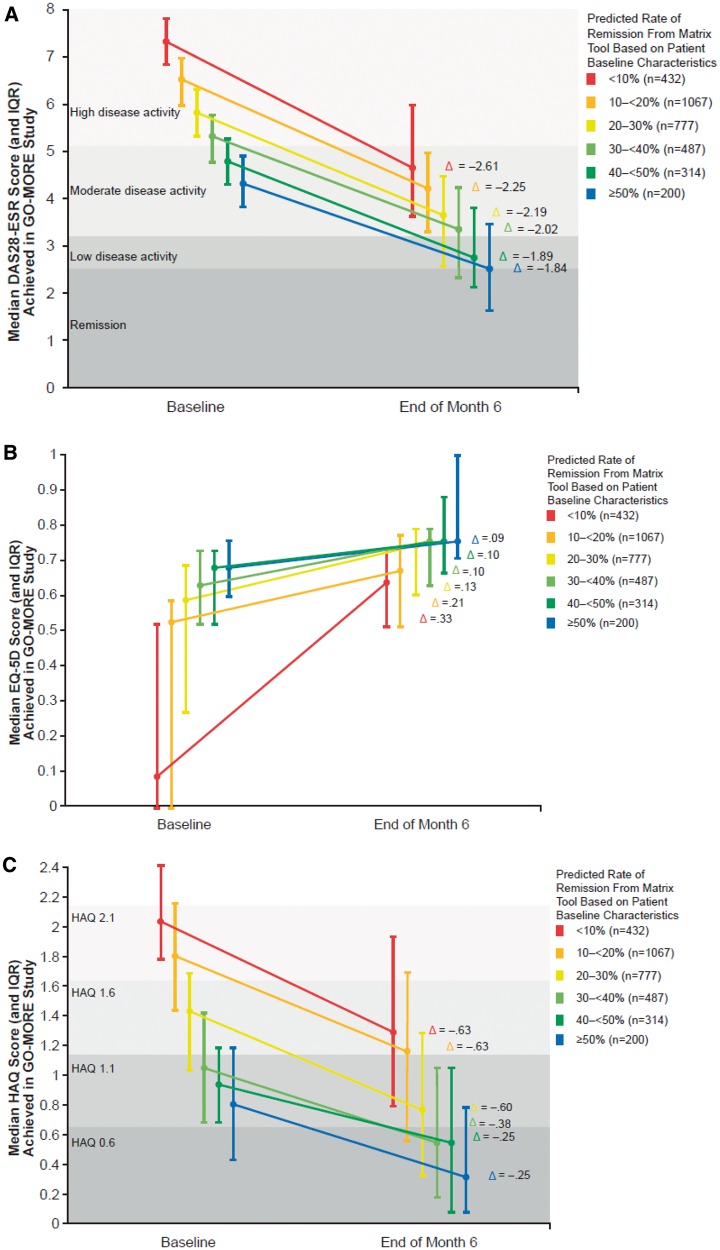
Relationship between predicted remission rate and other outcomes in the GO-MORE study The figure shows the relationship between the remission rate category predicted by the matrix model and the observed median DAS28 (**A**), EQ-5D (**B**) and HAQ (**C**) scores in the GO-MORE study. Figure 2A shows, for example, that for the patients who were predicted by the matrix tool to have a remission rate of < 10% (red line), the actual median DAS28 score at baseline was 7.34, with median improvement of 2.61 at month 6. For patients predicted by the matrix tool to have ≥50% remission rate (blue line), their median baseline DAS28 ESR score was 4.36, with median improvement of 1.84 at month 6. The vertical line indicates the interquartile range. Note that improvement is positive change for EQ-5D; improvement is negative change for DAS28 and HAQ. Δ: median change. EQ-5D, EuroQol 5-dimension; IQR, interquartile range.

In Fig. 2, patients are divided by their predicted rate of remission from the matrix tool, which is determined by their baseline characteristics. For each category of predicted remission (e.g. patients with 10% predicted rate of remission, shown in red in both the matrix model and Fig. 2), the median and interquartile range for baseline and month 6 DAS28-ESR are shown. The data reveal that patients who were predicted to have the lowest chance of remission (i.e. those in the <10% group; the red line in the figure, who also have the highest disease activity at baseline) also had the greatest change in DAS28-ESR score between baseline and month 6 **(**Fig. 2A). That is, the patients who had the worst values for their predictors improved the most and yet were still the least likely to attain remission. A similar pattern was seen for HAQ and EQ-5D scores (Fig. 2B and C).

**Fig. 3:**
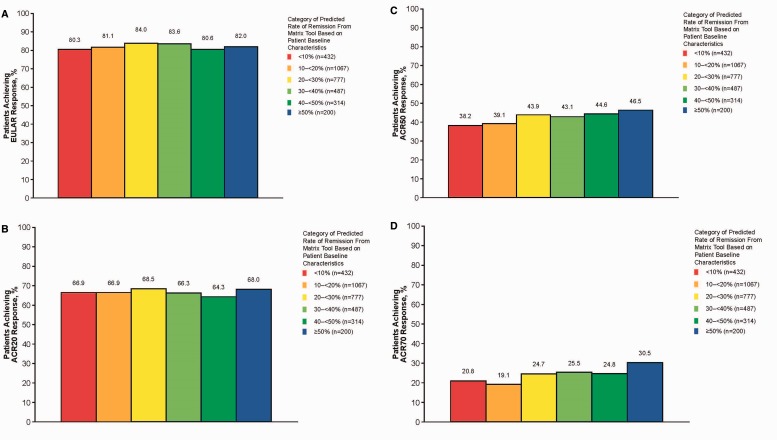
Relationship between predicted DAS28-ESR remission rate and EULAR response (A), ACR20 (B), ACR50 (C) and ACR70 (D) For each category of predicted DAS28-ESR remission rate from the matrix model, the figure shows the percentage of patients who attained good or moderate EULAR response after 6 months of GLM treatment. GLM: golimumab.

Figure 3 shows the relationship between the categories of predicted response rates derived from the matrix model and the actual month 6 attainment of EULAR response and ACR20, ACR50 and ACR70 response. EULAR response is a measure of both disease state and improvement over time, and it was not related to the predicted probability of remission. EULAR response was about 82%, regardless of the rate of remission predicted by the matrix tool. Similarly, no differences in ACR20 response rates were observed across different probabilities of remission; however, ACR50 and ACR70 response rates increased in patients with a higher predicted probability of remission (both P < 0.01).

## Discussion

Tools that determine which patients with RA might benefit from biologic treatment can help in making value-based decisions. We have developed a tool derived from data collected in a large trial of GLM treatment in more than 3000 patients with active RA despite DMARD treatment. The matrix tool predicts outcomes of 432 subpopulations of patients with RA identified by combinations of six characteristics (gender, HAQ, presence of comorbidities, age, TJC and ESR) that are associated with LDA and remission in the early treatment phase. The value of the model was not weakened by replacing ESR with CRP, which may be the preferred marker of inflammation used in clinical practice. It can assist clinicians in identification of patients for treatment, aid in establishing a treatment goal for an individual patient and provide guidance to policy-makers for the selection of RA patient populations that are likely to achieve good disease states from anti-TNF treatment. The model can be used to predict outcomes at several time points during treatment, and the predictions for disease activity are also relevant to physical function and quality of life outcomes. A similar matrix model approach to visualizing prediction of remission and response has been developed for patients with AS treated with biologics [14].

The data are important when considering the implications of treatment recommendations and reimbursement criteria on both patient selection criteria and patient outcomes as patients are considered for anti-TNF treatment. For example, in the UK, reimbursement for biologic treatment in RA is limited to patients who have a DAS28 >5.1. In Belgium, the threshold for reimbursement is DAS28 >3.7. The GO-MORE study included patients from both of these countries, and we compared treatment efficacy based on these eligibility criteria. For patients from the UK, 185/263 (70%) had DAS28-ESR >5.1 at baseline. For patients from Belgium, 114/123 (93%) had DAS28-ESR >3.7. In these subgroups of patients who would have been eligible for reimbursement, the selection criterion based on DAS28 led to large differences in the HAQ score, ESR and SJC of selected patients at baseline. As a result, remission was obtained by 20% of UK patients and 43% of Belgian patients. LDA was obtained by 35% of UK patients and 55% of Belgian patients. In this analysis, because of different policy regarding access to treatment in the UK and Belgium, the Belgian population had a doubled rate of remission.

Although patients with lower baseline disease activity were more likely to achieve remission and LDA, patients with high baseline disease activity demonstrated greater improvement in their DAS28, HAQ and EQ5D scores. The differences in remission rates and improvement rates in the different subpopulations counterbalanced each other and resulted in nearly equal EULAR response rates across the population (Fig. 3A). This may suggest that, at least for some DMARD refractory populations, response is a more appropriate goal of treatment than LDA or remission. At the same time, the excellent remission rates that can be obtained in patients with moderate disease activity, combined with ACR50 and ACR70 response rates (which are greater than those in patients with high disease activity), may be a convincing argument to open up that patient population for treatment in countries such as the UK.

Health Technology Assessments tend to be based on change-scores between two treatments compared in randomized, controlled trials and resulting in cost of quality-adjusted life-years. However, when clinicians make treatment choices or adjustments to treatments, they tend to rely on disease state rather than level of improvement. Elevated disease activity in patients with RA receiving anti-TNF treatment is associated with dose increases [15], and also the other direct and indirect costs of care of patients with RA are significantly lower in patients with LDA or remission as opposed to those who have moderate or high disease activity [16, 17]. Physical function, and to a lesser extent disease activity state, have been shown to be the major drivers of cost of RA management. Supplementary Tables S4 and S5, available at *Rheumatology* Online, summarize cost associated with different levels of HAQ, DAS28 and SDAI, and indicate that, even for biologic-treated patients, function and disease activity drive health care utilization costs of patients maintained on treatment. Therefore, not only the cost associated with change of disease state but also the disease state that is achieved is of importance for value-based decision making. Once the decision to treat is made, the eventual disease state achieved will be the driver of cost and further decision making. This may be an argument to value achievement of good disease state more than improvement. Another consideration for payers is that cost of treatment may be reduced if patients, as per EULAR recommendations, can taper or stop therapy after achieving sustained remission (EULAR recommendation 12) [12]. Better selection of patients who are likely to achieve remission may increase the likelihood of tapering or stopping therapy. Early achievement of remission appears to be an important component to successfully stopping therapy, which points to the relevance of the 1- and 3-month time points we chose for prediction of disease state in our analysis [18].

Most of the factors included in the matrix model are validated by a number of studies that have analysed individual predictors of RA treatment outcomes in studies of other biologics [1, 2], thereby increasing the face-validity of the model. Some characteristics, such as smoking, were not predictive in this dataset, but may be predictors in the overall RA population. The presence of comorbidities as a prognostic indicator has been shown previously [19], and it has also been shown that greater comorbidity is associated with greater physical disability in patients with RA [3]. Because collection of comorbidity data in GO-MORE was not rigorous, and comorbidities were likely to have been underreported in this study, future work should further explore the nature of the relationship between comorbidities and RA outcomes. In addition to the effect of comorbidities on outcomes, presence of certain serious comorbid diseases may considerably affect the treatment decision.

Further research will be helpful to validate this model in other populations, improve its predictive ability or expand its usefulness to include prediction of other outcomes. Although AUC in the ROC analysis of the prediction models was relatively high, not all factors that may affect response were included in the model. For example, patient expectations about effectiveness of treatment have been shown to be associated with remission [20]. The very large patient numbers are a clear strength of this analysis. A weakness of this model, however, is that the non-randomized nature of the study does not allow a direct comparison with patients who are not receiving an anti-TNF agent (e.g. in a placebo-controlled study) or are treated with an alternative medicine (e.g. DMARD combination therapy or a biologic with another mode of action). Although our model clearly highlights which RA subpopulations benefit more than other subpopulations during treatment with GLM, it does not address whether GLM is better than an alternative treatment strategy. Similar analyses in randomized studies could determine which treatment strategy is optimal in patient groups with characteristics that predicted the poorest outcomes in our study, such as older-aged female patients with high disease activity, severe disability and comorbidities.

### Conclusion

A matrix tool was developed to predict GLM treatment outcomes in patients with RA, based on a combination of six baseline demographic and disease characteristics of patients. Value of the outcome of therapy may be the amount of improvement in disease activity or the eventual disease state achieved. It is expected that such a tool will assist physicians, guideline committees and payers in providing practical guidance on identification and selection of appropriate candidates for anti-TNF therapy.

## Supplementary Material

Supplementary Data
