# Supplementary material for: Prediction of remission and low disease activity in disease-modifying anti-rheumatic drug-refractory patients with rheumatoid arthritis treated with golimumab
Source: Rheumatology (Oxford). 2016 Apr 25;55(8):1466–76. doi: 10.1093/rheumatology/kew179 (PMC4957672; doi:10.1093/rheumatology/kew179)
Supplement: Supplementary Data [file supp_55_8_1466__index.html]

Prediction of remission and low disease activity in disease-modifying anti-rheumatic drug-refractory patients with rheumatoid arthritis treated with golimumab — Prediction of remission and low disease activity in disease-modifying anti-rheumatic drug-refractory patients with rheumatoid arthritis treated with golimumab — Supplementary Data 

# Prediction of remission and low disease activity in disease-modifying anti-rheumatic drug-refractory patients with rheumatoid arthritis treated with golimumab

## Supplementary Data

files

- Supplementary Data - docx file
