# Supplementary material for: Prediction of remission and low disease activity in disease-modifying anti-rheumatic drug-refractory patients with rheumatoid arthritis treated with golimumab
Source: Rheumatology (Oxford). 2016 Apr 25;55(8):1466–76. doi: 10.1093/rheumatology/kew179 (PMC4957672; doi:10.1093/rheumatology/kew179)
Supplement: Supplementary Data [file supp_kew179_rhe-15-0896-File002.docx]

**SUPPLEMENTARY DATA**

**Supplementary table S1: Comorbidities reported in at least 2% of patients (N=3280)^a^**

| **System organ class** | **Preferred term** | **Number (%)** |
| --- | --- | --- |
| Blood and lymphatic system disorders | Anaemia | 113 (3.45) |
| Endocrine disorders | Hypothyroidism | 270 (8.23) |
| Gastrointestinal disorders | Gastritis | 156 (4.76) |
|  | Gastroesophageal reflux disease | 86 (2.62) |
|  | Dyspepsia | 72 (2.20) |
| Immune system disorders | Drug hypersensitivity | 112 (3.41) |
| Infections and infestations | Latent tuberculosis | 244 (7.44) |
| Metabolism and nutrition disorders | Hypercholesterolaemia | 266 (8.11) |
|  | Diabetes mellitus | 167 (5.09) |
|  | Hyperlipidaemia | 108 (3.29) |
|  | Dyslipidaemia | 103 (3.14) |
|  | Obesity | 87 (2.65) |
| Musculoskeletal and connective tissue disorders | Osteoporosis | 336 (10.24) |
|  | Osteoarthritis | 276 (8.41) |
|  | Osteopenia | 135 (4.12) |
|  | Spinal osteoarthritis | 96 (2.93) |
|  | Back pain | 74 (2.26) |
|  | Sjögren’s syndrome | 69 (2.10) |
| Psychiatric disorders | Depression | 197 (6.01) |
|  | Insomnia | 78 (2.38) |
| Respiratory, thoracic and mediastinal disorders | Asthma | 133 (4.05) |
| Social circumstances | Postmenopause | 89 (2.71) |
|  | Menopause | 84 (2.56) |
| Vascular disorders | Hypertension | 962 (29.33) |
|  | Varicose vein | 77 (2.35) |

^a^Patients were excluded from the trial for the following conditions: evidence of active tuberculosis or latent tuberculosis that is untreated; history of lymphoproliferative disease or any unknown malignancy or history of malignancy within the previous 5 years, with the exception of nonmelanoma skin cancer that has been treated with no evidence of recurrence; history of moderate to severe heart failure even if medically controlled; inflammatory rheumatic disease other than rheumatoid arthritis that might confound the evaluations of safety and toxicity such as, but not limited to, ankylosing spondylitis and psoriatic arthritis; any systemic inflammatory condition with signs and symptoms that might confound the evaluations of safety and toxicity from golimumab therapy, including, but not limited to: active Lyme disease, systemic lupus erythematosus, infectious or reactive arthritis, Reiter’s syndrome, nonrheumatoid vasculitis or parvovirus infection; allergy/sensitivity to investigational product(s) or its/their excipients, including latex; pregnant or intending to become pregnant; any clinically significant condition or situation, other than the condition being studied that, in the opinion of the investigator, would interfere with the trial evaluations or optimal participation in the trial.

**Supplementary table S2: Initial multivariate model predicting DAS28-ESR remission at Month 6 and low disease activity at Month 1**

| **Baseline variable** | **Wald Chi-square** | **P value** |
| --- | --- | --- |
|  | **Remission at Month 6** | |
| Gender | 13.1130 | 0.0003 |
| Smoking history | 7.0247 | 0.0298 |
| MTX category | 1.2557 | 0.7397 |
| HAQ category | 14.5097 | 0.0007 |
| Comorbidities | 9.4939 | 0.0021 |
| Age | 18.4272 | <0.0001 |
| TJC28 | 48.2602 | <0.0001 |
| SJC28 | 0.6006 | 0.4384 |
| Disease duration, years | 0.2943 | 0.5875 |
| Patient VAS | 2.5396 | 0.1110 |
| ESR (log) | 109.9716 | <0.0001 |
|  | **Low disease activity at Month 1** | |
| Gender | 9.3505 | 0.0022 |
| Age | 8.1473 | 0.0043 |
| MTX category | 2.1650 | 0.5389 |
| HAQ category | 6.2534 | 0.0439 |
| Comorbidities | 10.0289 | 0.0015 |
| TJC28 | 103.1994 | <0.0001 |
| SJC28 | 5.5348 | 0.0186 |
| Patient VAS | 9.8722 | 0.0017 |
| ESR (log) | 150.1696 | <0.0001 |

DAS28-ESR: disease activity score 28-erythrocyte sedimentation rate; HAQ,: health assessment questionnaire; MT:, methotrexate; SJC28: joint swollen joint count 28; TJC28: joint tender joint count 28; VAS: visual analogue scale.

**Supplementary table S3: Relationships between baseline predictor variables and 6-month outcomes of HAQ and EQ-5D scores**

|  | **Month 6 response**  **OR (95% CI)** | | |
| --- | --- | --- | --- |
|  | **EQ-5D** | | **HAQ** |
| **Baseline predictor variable** | **≥0.7** | **≥0.8** | **≤0.5** |
| ESR |  |  |  |
| <15 vs ≥45 | 1.06 (0.85–1.33) | 0.83 (0.65–1.08) | 0.80 (0.63–1.02) |
| ≥15 to <45 vs ≥45 | 0.98 (0.81–1.18) | 0.99 (0.80–1.22) | 0.80 (0.66–0.98) |
| Gender |  |  |  |
| Male vs female | 0.80 (0.65–0.99) | 0.96 (0.77–1.21) | 1.47 (1.19–1.81) |
| HAQ |  |  |  |
| 0 to 1.125 vs ≥1.75 | 5.86 (4.81–7.13) | 2.38 (1.91–2.96) | 6.55 (5.31–8.09) |
| ≥1.125 to <1.75 vs ≥1.75 | 1.62 (1.32–2.00) | 1.26 (0.99–1.60) | 1.60 (1.28–2.01) |
| Age, years |  |  |  |
| <35 vs ≥65 | 1.36 (0.99–1.87) | 1.36 (0.97–1.92) | 2.91 (2.06–4.12) |
| ≥35 to <50 vs ≥65 | 0.90 (0.71–1.15) | 0.94 (0.71–1.24) | 2.18 (1.65–2.87) |
| ≥50 to <65 vs ≥65 | 0.93 (0.75–1.17) | 0.94 (0.75–1.22) | 1.75 (1.35–2.27) |
| Comorbidities |  |  |  |
| No vs yes | 1.29 (1.06–1.56) | 1.63 (1.33–1.99) | 1.70 (1.39–2.07) |
| TJC |  |  |  |
| <10 vs ≥20 | 1.51 (1.19–1.92) | 1.44 (1.09–1.89) | 1.23 (0.96–1.59) |
| ≥10 to <20 vs ≥20 | 1.36 (1.09–1.71) | 1.21 (0.93–1.57) | 1.10 (0.86–1.39) |

CI: confidence interval; EQ-5D: EuroQol-5-dimension; ESR: erythrocyte sedimentation rate; HAQ: health assessment questionnaire; OR: odds ratio; TJC: tender joint count.

**Supplementary table S4: Mean annual cost per patient with rheumatoid arthritis by health assessment questionnaire score category**

| **HAQ score range** | **Sweden (n=183) [1]** None on biologic | | | **United Kingdom (n=916) [1]** None on biologic | | | **Canada (n=1086) [2]** On biologic | **HAQ score range** | **Germany [3]** | **Sweden [4]** | **Netherlands [5, 6]** |
| --- | --- | --- | --- | --- | --- | --- | --- | --- | --- | --- | --- |
|  | **Direct** | **Indirect** | **Total** | **Direct** | **Indirect** | **Total** | **Total** |  | **Sick leave** | **Disability (HCA)** | **Disability  (FCA)** |
| **<0.6** | $723 | $0 | $723 | $1,228 | $148 | $1,376 | $4,157 | **≤1.2** | € 856 | € 4,731 | € 752 |
| **0.6– <1.1** | $1,293 | $5,997 | $7,290 | $3,152 | $2,524 | $5,676 | $5,073 |  |  |  |  |
| **1.1– <1.6** | $1,924 | $8,524 | $10,448 | $2,091 | $3,474 | $5,565 | $5,645 | **1.2–1.7** | € 3,212 | € 12,707 | € 2,019 |
| **1.6– <2.1** | $3,672 | $15,588 | $19,260 | $3,087 | $5,300 | $8,387 | $9,861 |  |  |  |  |
| **2.1– <2.6** | $3,363 | $24,838 | $28,201 | $3,401 | $8,070 | $11,471 | $14,225 | **>1.7** | € 7,619 | € 18,894 | € 3,002 |
| **≥2.6** | $1,782 | $27,067 | $28,849 | $2,697 | $8,407 | $11,104 |  |  |  |  |  |

HAQ: health assessment questionnaire; FCA: friction cost approach; HCA: human capital approach.

**Supplementary table S5: Mean annual cost per patient with rheumatoid arthritis by disease activity level measured by DAS28 or SDAI**

| **Disease state** | **Mean (95% CI) total health care service utilization costs in  Canadian dollars** [7] 87% on biologic (total n=1086) | | **Mean costs (SD) in Euros** [8] 36.2% on biologic (total n=356) | | | |
| --- | --- | --- | --- | --- | --- | --- |
|  |  |  | **Resource use** | **Sick leave** | **Work disability (HCA)** | **Work disability (FCA)** |
|  | **DAS28** | **SDAI** | **SDAI** | **SDAI** | **SDAI** | **SDAI** |
| **Remission** | n=175 $3130 (2644–3617) | n=46 $2945  (1771–4120) | €828.28 (2,491.24) | €1,285.2 (1,502.1) | €5,772.8 (3,388.7) | €917.5  (538.3) |
| **Low disease activity** | n=911 $5992  (5333–6652) | n=1040 $5670  (5075–6266) | €1,039.02 (2,561.41) | €1,874.2 (2,185.9) | €7,186.6 (4,864.9) | €1,142.1 (772.8) |
| **Median or high disease activity** |  |  | €1,702.39 (3,500.74) | €3,291.9 (2,871.3) | €10,525.7 (6,129.2) | €1,672.5 (973.7) |

CI: confidence interval; DAS28-ESR: disease activity score 28-erythrocyte sedimentation rate; FCA: friction cost approach; HCA: human capital approach; SD: standard deviation; SDAI: Simple Disease Activity Index.

**Supplementary figure S1. Matrix model for prediction of 6-month DAS28-ESR LDA in male patients at 6-months (A) and DAS28-ESR LDA in male patients using CRP instead of ESR as a predictor (B).**

**A**


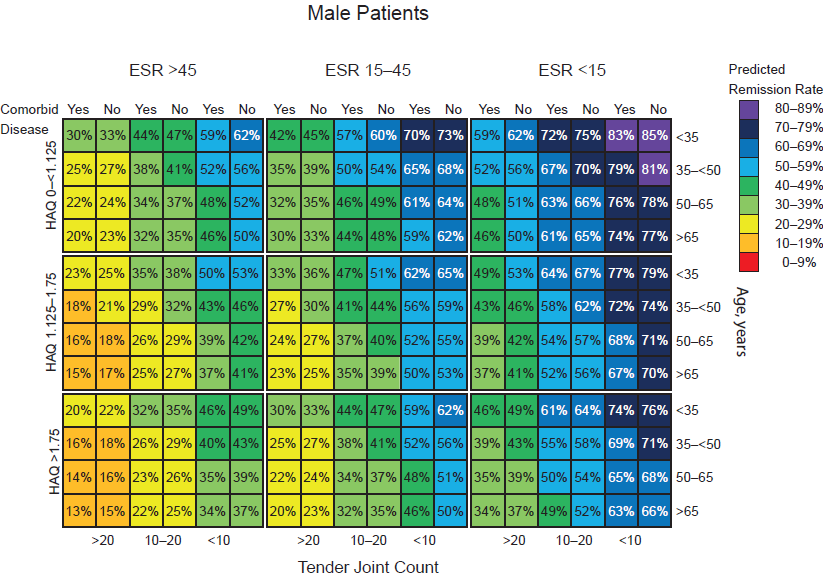


**B**


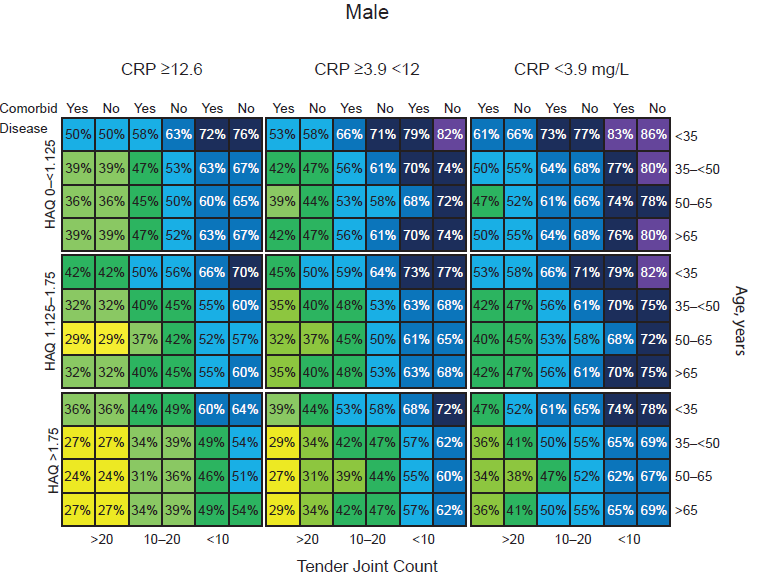


CRP: C-reactive protein; DAS28-ESR: disease activity score 28-erythrocyte sedimentation rate; HAQ: health assessment questionnaire; LDA: low disease activity; SDAI: Simple Disease Activity Index.

**References**

1. Kobelt G, Jönsson L, Lindgren P, [Young A](http://www.ncbi.nlm.nih.gov/pubmed/?term=Young%20A%5BAuthor%5D&cauthor=true&cauthor_uid=12355478), [Eberhardt K](http://www.ncbi.nlm.nih.gov/pubmed/?term=Eberhardt%20K%5BAuthor%5D&cauthor=true&cauthor_uid=12355478). Modeling the progression of rheumatoid arthritis: A two-country model to estimate costs and consequences of rheumatoid arthritis. Arthritis Rheum 2002;46:2310–9.

2. Ohinmaa AE, Thanh NX, Barnabe C, et al. Canadian estimates of health care utilization costs for rheumatoid arthritis patients with and without therapy with biologic agents. Arthritis Care Res (Hoboken) 2014;66:1319–27.

3. Huscher D, Merkesdal S, Thiele K, et al. Cost of illness in rheumatoid arthritis, ankylosing spondylitis, psoriatic arthritis and systemic lupus erythematosus in Germany. Ann Rheum Dis 2006;65:1175–83.

4. Johannesson M. The willingness to pay for health changes, the human-capital approach and the external costs. Health Policy 1996;36:231–44.

5. Brouwer WB, Koopmanschap MA. The friction-cost method: replacement for nothing and leisure for free? Pharmacoeconomics 2005; 23:105–11.

6. Koopmanschap MA, Rutten FF, van Ineveld BM, [van Roijen L](http://www.ncbi.nlm.nih.gov/pubmed/?term=van%20Roijen%20L%5BAuthor%5D&cauthor=true&cauthor_uid=10154656). The friction cost method for measuring indirect costs of disease. J Health Econ 1995;14:171–89.

7. Barnabe C, Thanh NX, Ohinmaa A, et al. Effect of remission definition on healthcare cost savings estimates for patients with rheumatoid arthritis treated with biologic therapies. J Rheumatol 2014;41:1600–6.

8. Radner H, Smolen JS, Aletaha D. Remission in rheumatoid arthritis: benefit over low disease activity in patient-reported outcomes and costs. Arthritis Res Ther 2014;21;16:R56.
